# A Novel Cognitive Function Scale Using Functional Near-Infrared Spectroscopy for Evaluating Cognitive Dysfunction

**DOI:** 10.3233/JAD-210072

**Published:** 2021-06-15

**Authors:** Shin Nakamura, Satoshi Yomota, Hitomi Ito, Nobuyuki Akinaga, Ayaka Hori, Kenta Chinomi, Hideaki Suzuki, Kazuhiko Uchida, Takashi Asada

**Affiliations:** a Analytical & Measuring Instruments Division, Shimadzu Corp., Kyoto, Japan; b Technology Research Laboratory, Shimadzu Corp., Kyoto, Japan; c Research Division, MCBI Inc., Ibaraki, Japan; d Faculty of Medicine, University of Tsukuba, Ibaraki, Japan; e Medical Corporation Association Sochikai, Memory Clinic Ochanomizu, Tokyo, Japan

**Keywords:** Cognitive dysfunction, functional neuroimaging, mental status and dementia tests, neuropsychological tests, neurovascular coupling, spectroscopy near-infrared

## Abstract

**Background:**

Maintaining cognitive function is integral to a healthy social life in the aged. Although neuropsychological tests and brain imaging methods can assess cognitive dysfunction, these techniques are subjective, psychologically burdensome, and cannot be conducted easily.

**Objective:**

We sought to develop an objective, low-burden novel cognitive function scale based on functional near-infrared spectroscopy (fNIRS) of hemodynamic changes in the cerebral cortex during daily task performance.

**Methods:**

A total of 63 participants (aged 60–80 years) identified as non-dementia controls (NDC) or with mild cognitive impairment (MCI) were recruited and randomly assigned to training and test data sets. Explanatory variables were hemodynamic responses during low-burden sensory and simple tasks without higher-order brain functioning.

**Results:**

A logistic regression analysis of the fNIRS index in NDCs and MCI patients revealed area under the curve, sensitivity, specificity, and holdout results of 0.98, 94%, 88%, and 62% respectively. Correlation between fNIRS index and MCI odds showed positive linearity (R^2^ = 0.96).

**Conclusion:**

Positive correlation between the fNIRS index and MCI odds indicated effectiveness of this fNIRS measurement. Although additional experiments are necessary, the fNIRS index representing degree of cognitive decline can be an onsite monitoring tool to assess cognitive status.

## INTRODUCTION

Cognitive function is integral to aging individuals maintaining a healthy social life. Daily evaluation can determine need for intervention. Currently, various conventional neuropsychological tests and brain imaging methods assess cognitive dysfunction. In order to assess the cognitive function of elderly who dislike taking tests using ordinary neuropsychological scales, it is important to be psychologically non-invasive. Widely used evidence-based psychological tests include Hasegawa’s Modified Mental State Examination and the Mini-Mental State Examination. However, these cognitive function tests impose psychological burdens on the subject, which may cause resistance to test especially among demented patients. Brain imaging methods including functional magnetic resonance imaging (fMRI) and positron emission tomography are used to detect early functional changes associated with early stage of cognitive decline [1]. Functional near-infrared spectroscopy (fNIRS) which allow brain measurements with few restrictions and does not require large-scale facilities may have potential application in dementia diagnosis [2].

Previous fNIRS studies have assessed cognitive status level in non-dementia controls (NDC) and patients with mild cognitive impairment (MCI) using forehead measurements [3–5]; however, these are significantly influenced by noise signals from skin blood flow [6, 7]. Diagnostic hemodynamic studies in patients with dementia have reported obtaining measurements during spatial cognitive tasks from the parietal lobe rather than the forehead [8, 9]. A more accurate cognitive function assessment would require simple tasks with less noise (e.g., body motion) while measuring the cerebral cortex region of interest (ROI) functionally related to the tasks.

Although the signal intensity average during the tasks is widely used to quantify task-induced hemoglobin change, some studies report using change rate or signal pattern as the feature quantity. For example, the feature quantity for psychiatric differentiation of mood disorders was increased oxygenated hemoglobin (Oxy-Hb) rate in the prefrontal area during the verbal fluency task, and the signal waveform centroid value [10, 11]. This practice demonstrates that psychiatric fNIRS applications have employed features other than the general signal intensity average.

We aimed to index degree of cognitive decline between NDC and MCI patients through logistic regression analysis using the fNIRS waveform centroid value to develop a cognitive function evaluation system for objective, easy, and low-burden routine measurements. We evaluated clinical efficacy of the fNIRS index by validating its discriminative power between NDC and patients with MCI, as well as index performance based on test data.

## MATERIALS AND METHODS

### Participants

In this observational study we enrolled healthy volunteers, and outpatients and their partners aged between 60 and 80 years. All participants provided written informed consent. This study was approved by the Ethical Committee for human research at the Memory Clinic and was performed according to the following criteria: MCIs were diagnosed by Petersen’s criteria [12]; NDCs matched neither Petersen’s MCI nor dementia definition in Diagnostic and Statistical Manual of Mental Disorders (Major Neurocognitive disorder; American Psychiatric Association) [13], and completed by a psychiatric doctor’s comprehensive judgment with a diagnosis confidence level (1: No confidence, 2: A little confidence, 3: Moderate confidence, and 4: High confidence).

**Fig. 1:**
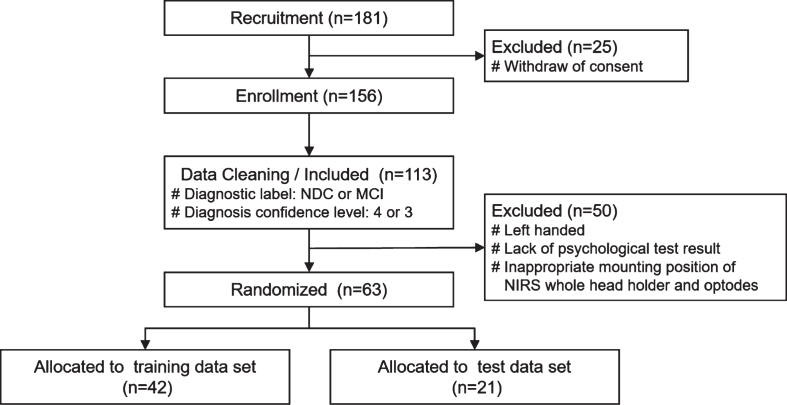
Flowchart representing the study participants and data cleaning. 181 healthy volunteers, outpatients and their partners aged between 60 and 80 years were recruited and diagnosed in the Memory Clinic. After fNIRS measurement of enrolled 156 participants, 63 subject data were selected according to the inclusion and exclusion criteria, and randomly assigned in a ratio of 2:1 to two data sets as follows: 42 to the training data set and 21 to the test data set. MCI, mild cognitive impairment; NDC, non-dementia control.

**Fig. 2:**
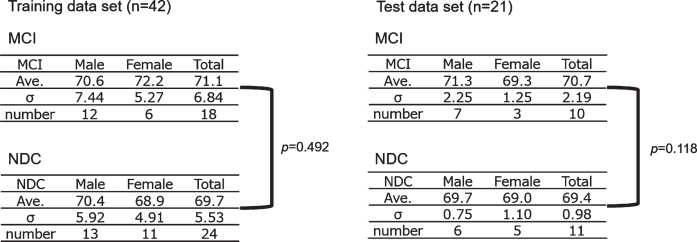
Subject allocation. The gender and age in the MCI and NDC group were shown. Neither the training data set nor the test data set showed a significant difference in age between the MCI and NDC groups. MCI, mild cognitive impairment; NDC, non-dementia controls; Ave., average age.

We recruited 181 participants and selected subject data according to the following inclusion and exclusion criteria. Inclusion criteria were 1) Patients with MCI and NDCs, and 2) Diagnosis confidence level 4 and 3; exclusion criteria were 1) Being left-handed, 2) Non-availability of complete Mini-Mental State Examination, Clinical Dementia Rating, Wechsler Memory Scale-Revised, and Geriatric Depression Scale results, and 3) Inappropriate mounting position of fNIRS whole head holder and optodes. After the application of these criteria, data from 63 (N = 63) subjects were finally included and randomly assigned in a ratio of 2:1 to two data sets: 42 to the training data set and 21 to the test data set (Fig. 1). Clinical characteristics of the participants are summarized in Table 1 and Fig. 2.

**Table 1:** Clinical characteristics of the participants

Subject characteristics	MCIs	NDCs	p
N (female + male)	28 (9 + 19)	35 (16 + 19)
Age, range / mean (SD)	60–79 / 71.0 (5.7)	61–80 / 69.6 (4.7)	0.31
Years of education, mean (SD)	14.6 (2.0)	14.5 (2.1)	0.91
MMSE, mean (SD)	26.9 (2.1)	28.9 (1.3)	< 0.01
WMS-R Immediate, mean (SD)	8.7 (3.9)	12.2 (3.9)	< 0.01
WMS-R Delayed, mean (SD)	6.4 (4.9)	10.1 (4.9)	< 0.01
CDR, mean (SD)	0.5 (0.4)	0.0 (0.1)	< 0.01
GDS, mean (SD)	2.1 (1.7)	1.9 (2.2)	0.70

MCIs, mild cognitive impairment; NDCs, non-dementia controls; SD, standard deviation; MMSE, Mini-Mental State Examination; WMS, Wechsler Memory Scale; CDR, Clinical Dementia Rating; GDS, Geriatric Depression Scale.

### Measurement tasks and execution methods

In our previous study, we used simple low-burden sensory tasks elderly demented individuals could perform at daily functioning level without higher-order brain function. In this study, we employed a working memory assessment task (modified serial number task) involving calculations, and a skin tactile sensation assessment task (SUMANU task) [14]. To accurately capture task-induced brain activity involving working memory and sensory functions, both tasks were performed in a static sitting position with restricted body movements during the fNIRS measurement to avoid noise associated with body movements.

**Fig. 3:**
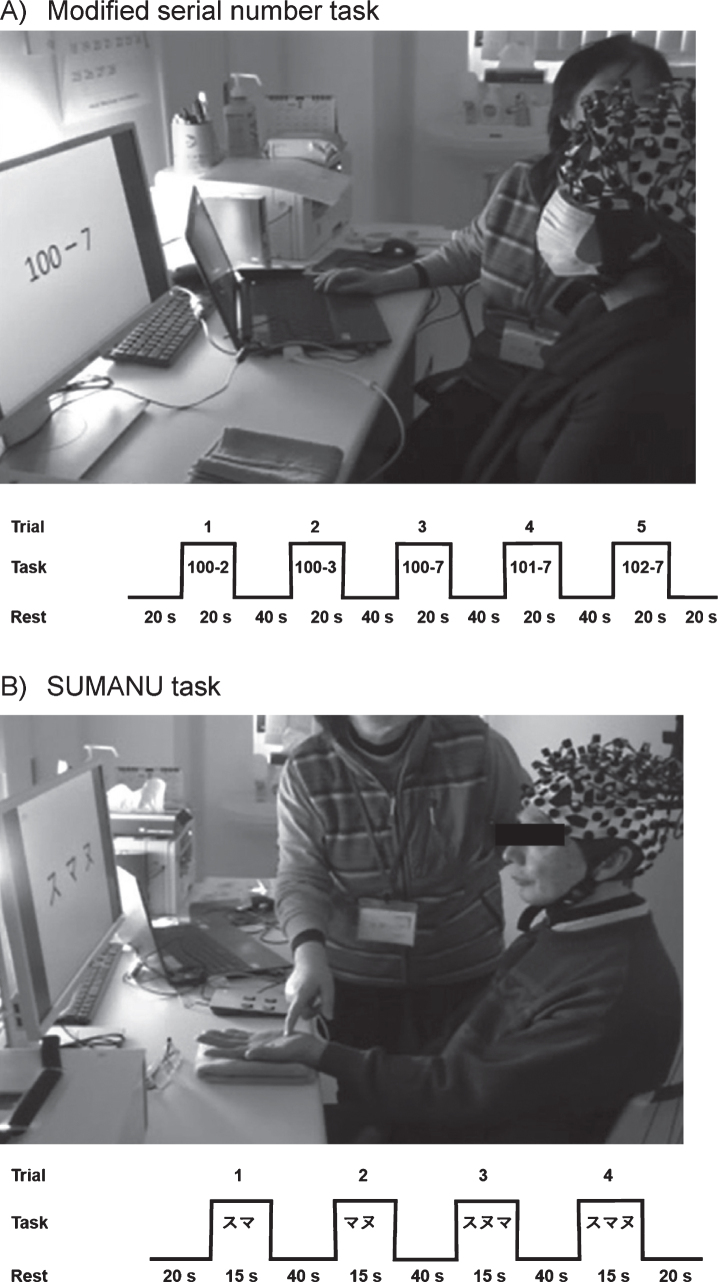
Functional near-infrared spectroscopy measurement tasks. A) Modified serial number task: A total five continuous subtraction questions were presented on the monitor. The participants watched the questions on the monitor to recognize a subtraction problem and calculated the answer while looking at the problem. They remembered the difference from the last problem and did the next equation. During a 40 s rest interval in between each task, participants were asked to pronounce meaningless vowels (a, i, u, e, o) to determine the utterance-associated signal components in the baseline signal. B) SUMANU task: The participants closed their eyes and the instructor drew the Japanese katakana characters “
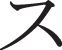
(SU),” “
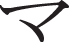
(MA),” and “
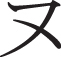
(NU)” on the palm of the participant’s left hand within 15 s. The participants remembered the linguistic characters with similar shapes drawn on the left palm with their eyes closed. Then, they recalled the letters at the end of the task period. The task was performed four times after 20 s resting intervals with two instances involving two-character drawings and two instances involving three-character drawings.

In the modified serial number task, we presented a math problem on the monitor in front of participants requiring continuous subtractions to obtain the answer. Each of the following subtractions—100-2, 100-3, 100-7, 101-7, and 102-7—was performed for 20 seconds. A total of five problems were presented with increasing difficulty. The participants read and calculated answers to the problems while viewing the monitor. This was repeated until the end of the task period. During a 40-s rest interval in between each task, participants were asked to pronounce meaningless vowels (*a*, *i*, *u*, *e*, *o*) to determine utterance-associated signal components in the baseline signal (Fig. 3A).

In the SUMANU task, participants closed their eyes to remove vision-associated disturbance and the instructor drew the Japanese katakana syllabary “
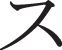
(*SU*),” “ 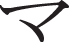
(*MA*),” and “ 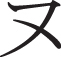
(*NU*) “ on the palm of the participant’s left hand within 15 s. Subsequently, the participant was asked to guess the character. The task was performed four times after 20 s resting intervals with two instances involving two-character drawings and two instances involving three-character drawings (Fig. 3B). A total of four questions were presented with increasing difficulty. Participants then remembered characters drawn on the left palm with their eyes closed. Then, they recalled the letters at the end of the task period.

Noise suppression, including increased heartbeat due to extreme tension or impatience, was achieved by conducting the tasks at a low degree of difficulty before data acquisition. Measurements were performed after ensuring participants fully understood how to conduct the tasks.

### fNIRS measurement

**Fig. 4:**
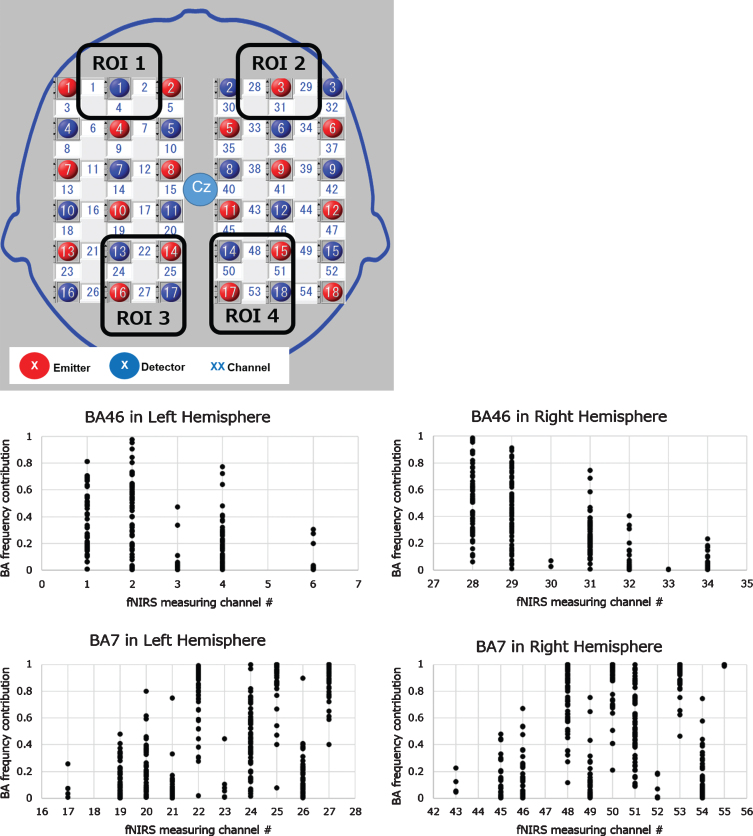
Optodes allocation, target ROI, and channels. A lattice arrangement of 18 pairs of optodes provided 54 channels to measure a whole head region. After fNRIS measurement and 3D digitizing of optode positions, the Brodmann area (BA) contribution rate at each channel of all participants from the digitized optode positions data was calculated. The target channels at the target ROIs defined by the 2×2 matrix square of the NIRS measurement area were selected referring to the distribution of the target BA contribution. The target BAs were the dorsolateral prefrontal cortex (DLPFC; BA46) for the modified serial number task and the somatosensory cortex (SC; BA7) for the SUMANU task. ROI, region of interest.

We acquired fNIRS data using a continuous 3-wavelength fNIRS LABNIRS 67ch system with a whole head holder (Shimadzu, Japan). We arranged optodes that provided 54 channels to measure not only the forehead region but also the dorsolateral prefrontal region, parietal lobe, and somatosensory region using the following locations of the 10–20 system: P3, P4, F3, and F4 coordinates with the time-divided method (33 ms/point) (Fig. 4). We digitized each participant’s channel position using the Fastrak 3D digitizer (Polhemus, US) with input to the standard brain model and Montreal Neurological Institute coordinate outputs using the NIRS-SPM software Ver. 4 (Department of Bio and Brain Engineering, KAIST, Korea) to obtain the brain anatomical information from the measurement channel.

### Feature quantity

#### Target region of interest and channels

We calculated the Brodmann area (BA) contribution rate of all participants at each channel from the Montreal Neurological Institute coordinate outputs to analyze the hemodynamic changes related to the tasks. Target ROI and channels were decided following the steps elaborated below. The modified serial number task could assess working memory activities and the SUMANU task could assess sensory activities; hence, we set the dorsolateral prefrontal cortex (DLPFC; BA46) for the modified serial number task and the somatosensory cortex (SC; BA7) for the SUMANU task as target BA. We analyzed the frequency distribution of both the BA46 and BA7 contribution rate to each channel of all participants. Our goal was to develop a simple, easy-to-use, and affordable wearable device that could provide new cognitive function scale. We set up to four channels at each ROI defined by the 2×2 matrix square of the NIRS measurement area designed by including two light transmitters and two detectors, and selected target channels with higher target BA frequency distributions (Fig. 4).

#### Hemodynamic feature quantity

The Oxy-Hb waveform obtained from the fNIRS signal underwent a discrete wavelet transformation (Daubechies) to extract the hemodynamic signal with neurovascular coupling frequency (0.05–0.2 Hz) and remove other signal frequency caused by noise or artifacts [15] (Supplementary Figure 1). We calculated the task signal as degree of change from the pre-task signal to eliminate influence of a long fluctuation period. We calculated the task signal mean value by subtracting the baseline signal during 5 s of the pre-rest period from the signal area during the task period; furthermore, we calculated the centroid value of the task signal as normalized value with the task period set to 1 from the start of each task.

The dynamic range or response of blood flow changes might vary across individuals due to differences in skull size and thickness in the measurement area and structural characteristics of the blood vessels. We assumed that using the ratio of the second and third features from the repetitive tasks, while altering the difficulty degree, could correct these between-subject variations. We calculated 28 features for every two tasks using signals obtained from the 14 channels.

### Regression analysis

To quantify the cognitive decline degree in the NDC and MCI groups, we used the training data shown in Fig. 2 and Table 1 to perform a ridge regression analysis with the objective variable NDC = 0/MCI = 1 while adding all the explanatory variables to the regression equation (Python ver. 3.6.3/scikit-learn = 0.19.1). We cross-validated the regression models five times, and assessed the clinical efficacy using receiver operating characteristic (ROC) analysis. Moreover, we performed a holdout evaluation to determine the predictive ability of the cognitive decline degree obtained from the regression model using the test data.

## RESULTS

We obtained the fNIRS index (equation A) through regression analysis. This logistic regression model involved weighing the Oxy-Hb features of the two tasks as explanatory variables for each fNIRS measurement area of 14 channels (see Supplementary Material 1).

(A) $$P=1/-\left\{1+\exp\left(-\alpha +\sum_{n=1}^{28}CnFn\right)\right\}$$

P, fNIRS index; *α*, Constant; Cn, Coefficient of the explanatory variable; Fn, Feature quantity of the fNIRS signal

**Fig. 5:**
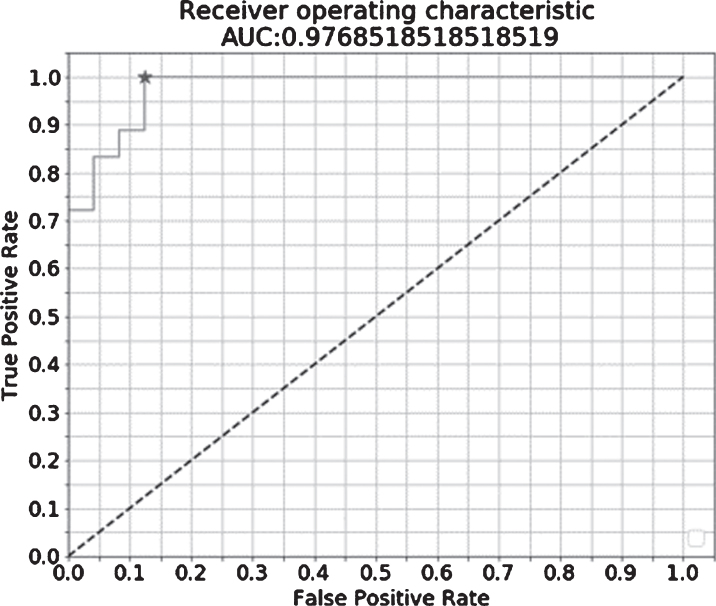
Receiver operating characteristic analysis of regression model between non-dementia controls and patients with mild cognitive impairment. After a ridge regression analysis of training data with the feature quantities of cerebral hemodynamic change related to two tasks as the explanatory variable and NDC = 0/MCI = 1 as the objective variable, the false positive rate on the horizontal axis and the true positive rate on the vertical axis was plotted. The result was AUC 0.977 and cut-off 0.417. AUC, area under the curve.

**Fig. 6:**
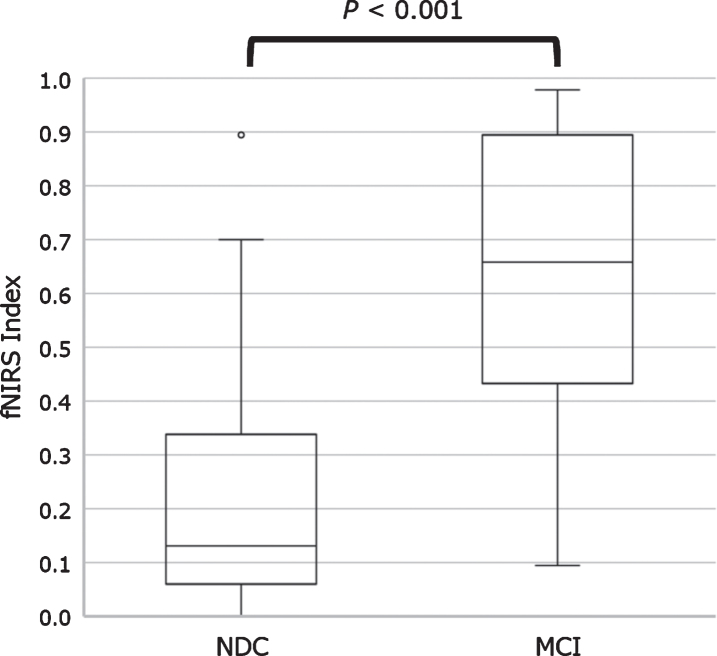
Boxplot representing functional near-infrared spectroscopy index values between NDC and patients with MCI. The fNIRS index was calculated as a numerical value from 0 to 1 through regression analysis. Boxplot of fNIRS index showed a significant difference in the NDC and MCI groups. MCI, mild cognitive impairment; NDC, non-dementia controls.

**Fig. 7:**
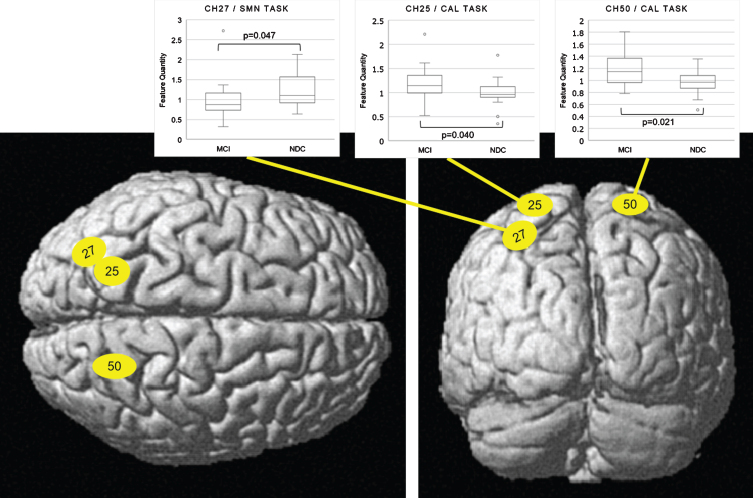
Anatomical location of channels with the features showing a significant difference (Left: top view, Right: rear view). Channel location was calculated and mapped on the normalized brain surface using NIRS-SPM software Ver. 4 (Department of Bio and Brain Engineering, KAIST, Korea), MATLAB software R2013b (Math Works, US), and SPM8 software (Wellcome Department of Cognitive Neurology, UCL, UK). CH27 in SC (BA7) in the SUMANU task and CH25/CH50 in SC (BA7) in the modified serial number task showed significant differences (see boxplot of the feature quantities). All three channels were in the parietal cortex region on the standard brain calculated by average Montreal Neurological Institute coordinates of the participants. CH 27, channel number 27; CH 25, channel number 25; CH50, channel number 50; SMN TASK, SUMANU task; CAL TASK, modified serial number task.

**Fig. 8:**
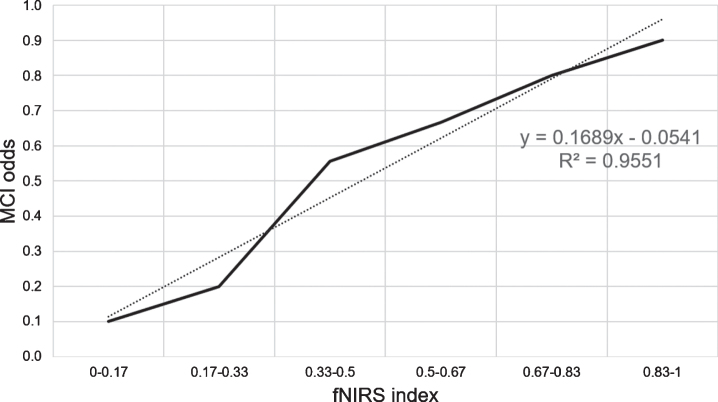
Correlation between fNIRS Index and MCI odds. The abundance ration of MCI was calculated in the even section of fNIRS index divided between 0 and 1 into six equal intervals. The fNIRS index showed a high positive correlation with the MCI odds (R^2^ = 0.96). fNIRS, functional near-infrared spectroscopy; MCI, mild cognitive impairment.

There was a significant difference in the fNIRS index between the NDC and MCI groups in the training data set (*p* < 0.001). ROC analysis revealed area under the curve, accuracy, cross-validation accuracy (mean), sensitivity, and specificity of 0.98, 91%, 50%, 94%, and 88%, respectively (Fig. 5). The holdout evaluation of the test data set showed accuracy, sensitivity, and specificity of 62%, 50%, and 73%, respectively. The boxplot analysis showed no large variation in the index distribution in the test data set; furthermore, we confirmed a significant between-group difference in the fNIRS index (*p* < 0.001) (Fig. 6). The feature quantities of each channel in both task measurements were tested for significant differences using Wilcoxon’s rank-sum test in the NDC and MCI groups. Figure 7 shows the *p*-value of measurement channels satisfied with a significance level of 5% or less. There were significant differences at channel number 25 (CH25) and CH50 in the modified serial number task and CH27 in the SUMANU task. Finally, we equally divided the fNIRS index of all participants into six intervals (0–0.17; 0.17–0.33; 0.33–0.5; 0.5–0.67; 0.67–0.83; and 0.8–1) and calculated the abundance ratio of MCI in each interval population (MCI odds). The fNIRS index showed a high positive correlation with the MCI odds (R^2^ = 0.96) (Fig. 8).

## DISCUSSION

We aimed to develop a new, objective cognitive function scale utilizing information directly from low-burden brain activities. Two tasks—math calculation and short-term memory—activated ROIs related to the dorsolateral prefrontal and somatosensory cortex. Functional brain imaging modality fNIRS visualized hemodynamic changes in Oxy-Hb. Next, we used logistic regression analysis to develop a novel index of the cognitive decline degree in NDC and MCI groups with the feature hemodynamic changes as explanatory variable. Resulting regression models discriminated between the NDC and MCI groups, and the fNIRS Index *P* (Eq. (A)) showed significantly positive linearity with MCI odds. This suggested the fNIRS Index could be a new functional scale reflecting cognitive decline.

ROC analysis of the training data set showed area under the curve, total accuracy, cross-validation accuracy, sensitivity, and specificity of 0.98, 91%, 50%, 94%, and 88%, respectively. The holdout result was 62%, while sensitivity for discriminating MCI was low; however, specificity was high. Despite relatively low sensitivity, we found a highly positive correlation between the fNIRS index and the MCI odds. This indicated the fNIRS index is a sufficiently effective cognitive scale to monitor cognitive decline risk. The Oxy-Hb signal waveform centroid value during the task period indicating initial rise of hemodynamics during brain activation was the feature quantitative variable of interest. Results ratio of second to third iterations of each increasingly difficult task was seen to demonstrate adaptability to task difficulty level.

The discriminating power of the training data with similar features using Deoxy-Hb instead of Oxy-Hb, which has a negative correlation with Oxy-Hb in activation derived from brain function, was area under the curve > 0.9 (data not shown). An activation signal derived from neurovascular coupling was extracted optimally by wavelet filtering, and ratio of the second and third features from repetitive tasks normalized the blood flow dynamics for each individual and reduced heterogeneity among participants. Accordingly, fNIRS waveform characteristics could ensure consistency with neurovascular coupling theory during brain activation, and results of both Oxy-Hb and Deoxy-Hb were discriminating.

Interestingly, both NDC and MCI groups showed no significant difference in DLPFC (BA46) involving short-term memory when executing both tasks. In contrast, results indicated significant differences at CH27 in SC (BA7) in the SUMANU task, and at CH25 and CH50 in SC (BA7) in the modified serial number task. Average contribution rate of BA7 at CH25, CH27, and CH50 in training data set participants was 90% or more. From an anatomical point of view, BA7 includes the superior parietal lobule (SPL), inferior parietal lobule (IPL), and temporo-parietal junction (TPJ) across the intraparietal sulcus (IPs). As shown in Fig. 7, we found that CH25 and CH50 were in SPL and IPs, and CH27 was in IPL, IPs, and TPJ on the standard brain calculated by average Montreal Neurological Institute outputs. Previous studies related to attention control in visual stimuli using fMRI and animals reported that SPL, IPs, IPL, and TPJ were responsible for the interaction mechanism between the dorsal attention network, which is an active attention control network, and the passive ventral attention network [16].

Performance of both tasks showed no significant differences between groups: Total number of answers in the modified serial number task were MCI 34.1±*σ*13.3 versus NDC 36.1±*σ*10.0, *p* = 0.51. SUMANU task total correct answer rate was MCI 75.4±*σ*25.9% and NDC 80.6±*σ*13.2%, *p* = 0.34. This indicated there may no appreciable difference in executive functions for both tasks in NDC and MCI groups since higher-order brain function capacity was not required. Accordingly, there was no difference in hemodynamic response in a wide range of BA46 channels for working memory, nor in BA7 for left hand somatosensory function.

In contrast, attention control in both groups while executing both tasks registered different hemodynamic responses associated with interaction between dorsal and ventral attention networks. The modified serial number task required recalling previous answers and repeating subtraction while viewing subtraction problems on the monitor. That is, visual stimuli were constantly present in this task. The SUMANU task required participants remember and verbally identify characters drawn on the left palm with eyes closed and later recall the image. There was no direct visual stimulus, but spatial attention and linguistic cognition was elicited. These differences in attention control during tasks may affect channel position and signaling features between tasks. We also found significant differences between two tasks in areas anatomically important for attention control networks: SPL, IPL, IPs, and TPJ. Hemodynamic response may be different between NDC and MCI groups due to adapting attention control for increasing difficulty regardless of task. This hypothesis needs to be validated through further study with other modalities such as fMRI.

We used the novel fNIRS index to evaluate significant between-group differences in cognitive processes, including working memory or sensory function. However, this study has some limitations. First, size of NDC and MCI groups was limited. As we enrolled participants from a single memory clinic, there may be some bias in the sample. Second, the quantity of interest consisted of hemodynamic changes from several regions of the cerebral cortex but did not involve any information from the limbic cortex, which has an important role in memory, emotional activity, and independent nervous activity. Since contraction and amyloid-*β*aggregation in the cerebral cortex are important in Alzheimer’s disease pathology, cerebral hemodynamic changes may potentially be affected by disease lesions. Unfortunately, pathological evidence was not part of this study. Third, the fNIRS index was calculated with a regression analysis of the NDC and MCI groups; however, both NDC and MCI populations were heterogeneous. In addition, fNIRS index availability for personal time course scale of cognitive change was not clear.

In future, longitudinal studies tracking pre-post intervention cognitive status of individuals compared to MCI/dementia prevention interventions assessed by conventional tests could verify convergent validity of the fNIRS index. It is also necessary to assess comprehensive cognitive function indices in combination with indices of daily life activity. To contribute to the social implementation of dementia prevention interventions for quality of life improvement of patients and their families in this aging society, we plan to carry out a future study to confirm validity and robustness of this fNIRS measurement in a multi-institutional or cross-sectional longitudinal intervention study for MCI and pre-MCI individuals.

Although early in development, the novel cognitive function scale described here involving the fNIRS index could calculate degree of cognitive decline degree in NDCs and MCI patients. Our method of cognitive function assessment based on cerebral hemodynamic response to daily function tasks provided an objective and low psychological burden measurement of cognitive decline in contrast with subjective psychological tests. Moreover, large-scale equipment or physical restraints were not required in our assessment unlike in conventional brain imaging methods. In the future, a task-related, automated fNIRS measuring system could provide a novel index for monitoring degree of cognitive decline in daily life, could be performed without a clinical psychologist to further complement conventional psychological tests.

## Supplementary Material

Supplementary Material
