# Supplementary material for: A Novel Cognitive Function Scale Using Functional Near-Infrared Spectroscopy for Evaluating Cognitive Dysfunction
Source: J Alzheimers Dis. 2021 Jun 15;81(4):1579–88. doi: 10.3233/JAD-210072 (PMC8293658; doi:10.3233/JAD-210072)
Supplement: Supplementary Material [file jad-81-jad210072-s001.pdf]

Supplementary Material

A Novel Cognitive Function Scale Using Functional Near-Infrared Spectroscopy for Evaluating Cognitive Dysfunction

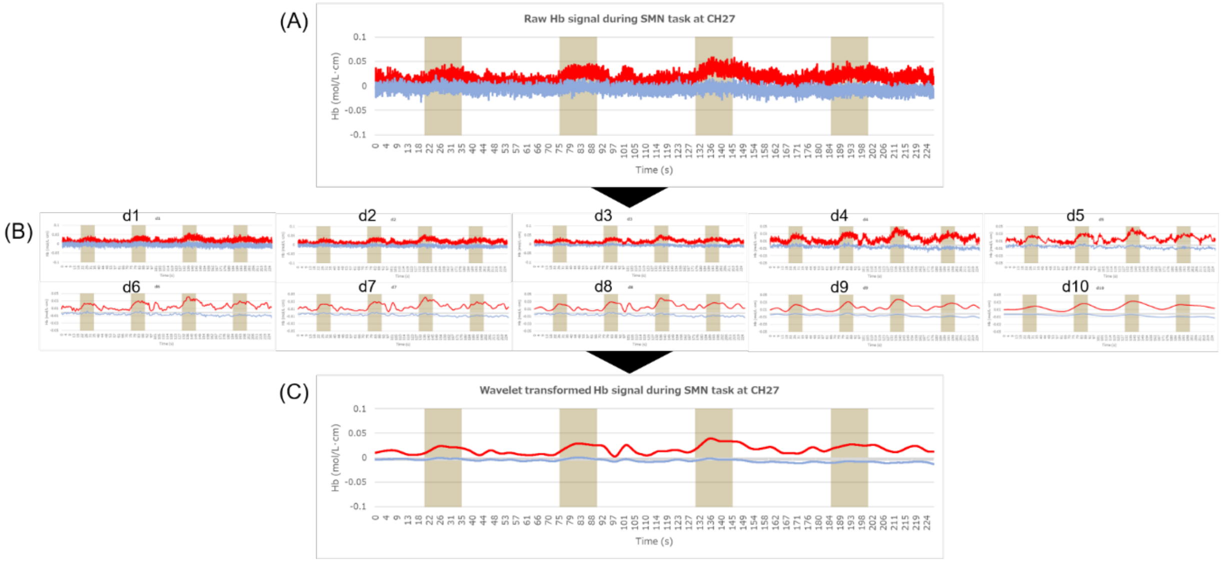

Supplementary Figure 1. Wavelet filtering: Hb signal (Oxy-Hb; red line, Deoxy-Hb; blue line) during the task. Colored time zones show the repeated task period. A) Raw Hb signal, B) Raw Hb signal was decomposed discrete oscillations in ten stages by Daubechies wavelet (d1-d10). C) We selected d9 oscillation since it was similar to the oscillation of the task period time (0.05-0.07 Hz). Wavelet transformed Hb signal was reconstructed using d9 including neighbor d8 and d10 to remove various artifacts like breathing (0.15-0.5 Hz), heartbeat (0.5-2 Hz), and spike noise or system white noise (>2 Hz).

Supplementary Material 1. Formula details of Eq. (A)

$$P = 1/\ \{1 + \exp(-\alpha + \sum_{n=1}^{28} CnFn)\} \qquad \text{Eq.(A)}$$

P: fNIRS Index, α: Constant, Cn: Coefficient of the explanatory variable, Fn: Feature quantity of the fNIRS signal

α=-0.3828

C1= 0.5983, C2= 0.2925, C3= 1.0099, C4= 0.5093, C5= 0.2998, C6= 0.1907, C7= -0.0895, C8= -0.3086, C9= -0.6974, C10= -0.2771, C11= -0.5422, C12= 0.9944, C13= -0.5590, C14= -0.0467, C15= 0.2068, C16= -0.4786, C17= -0.1670, C18= -0.0077, C19= -0.0462, C20= -0.5857, C21= -0.5101, C22= -0.2794, C23= 0.0588, C24= 0.3140, C25= -0.3180, C26= 0.0403, C27= -0.4997, C28= 1.4226

F1: CAL/CH1, F2: CAL/CH2, F3: CAL/CH4, F4: CAL/CH22, F5: CAL/CH24, F6: CAL/CH25, F7: CAL/CH27, F8: CAL/CH28, F9: CAL/CH29, F10: CAL/CH31, F11: CAL/CH48, F12: CAL/CH50, F13: CAL/CH51, F14: CAL/CH53, F15: SMN/CH1, F16: SMN/CH2, F17: SMN/CH4, F18: SMN/CH22, F19: SMN/CH24, F20: SMN/CH25, F21: SMN/CH27, F22: SMN/CH28, F23: SMN/CH29, F24: SMN/CH31, F25: SMN/CH48, F26: SMN/CH50 , F27: SMN/CH51 , F28 SMN/CH52

CAL: Modified serial seven task, SMN: SMNANU task, CHXX: measuring channel number

Supplementary Table 1. Diagnosis confidence criteria and result

Diagnosis confidence level	Criteria	Number of NDC	Number of MCI
**4** High confidence	All diagnostic information available	43	27
**3** Moderate confidence	MRI finding was not typical	17	26
**2** A little confidence	MRI was not available	1	0
**1** No confidence	Neither MRI nor knowledgeable informant was not available	0	0

NDC was judged comprehensively by conducting a series of psychological tests (MMSE, WMS, CDR, GDS; see Table 1) and clinical interviews by doctors [17]. Alzheimer's disease likelihood was estimated by the degree of atrophy in the parahippocampal gyrus as VSRAD score statistically calculated by Voxel-based Specific Analysis System for Alzheimer's disease [18]. Typically, VSRAD score of NDC is 0 to 1 and the one of MCI or dementia is 1 to 3.
